# Generation and Characterization of a Zebrafish Model for *ADGRV1-*Associated Retinal Dysfunction Using CRISPR/Cas9 Genome Editing Technology

**DOI:** 10.3390/cells12121598

**Published:** 2023-06-10

**Authors:** Merel Stemerdink, Sanne Broekman, Theo Peters, Hannie Kremer, Erik de Vrieze, Erwin van Wijk

**Affiliations:** 1 Department of Otorhinolaryngology, Hearing & Genes, Radboud University Medical Center, 6525 GA Nijmegen, The Netherlands; 2 Donders Institute for Brain, Cognition and Behaviour, Radboud University Medical Center, 6525 GA Nijmegen, The Netherlands; 3 Department of Human Genetics, Radboud University Medical Center, 6525 GA Nijmegen, The Netherlands

**Keywords:** *ADGRV1*, Usher syndrome, CRISPR/Cas9, zebrafish, retinal dysfunction, retinitis pigmentosa

## Abstract

Worldwide, around 40,000 people progressively lose their eyesight as a consequence of retinitis pigmentosa (RP) caused by pathogenic variants in the *ADGRV1* gene, for which currently no treatment options exist. A model organism that mimics the human phenotype is essential to unravel the exact pathophysiological mechanism underlying *ADGRV1-*associated RP, and to evaluate future therapeutic strategies. The introduction of CRISPR/Cas-based genome editing technologies significantly improved the possibilities of generating mutant models in a time- and cost-effective manner. Zebrafish have been recognized as a suitable model to study Usher syndrome-associated retinal dysfunction. Using CRISPR/Cas9 technology we introduced a 4bp deletion in *adgrv1* exon 9 (*adgrv1^rmc22^*). Immunohistochemical analysis showed that Adgrv1 was absent from the region of the photoreceptor connecting cilium in the *adgrv1^rmc22^* zebrafish retina. Here, the absence of Adgrv1 also resulted in reduced levels of the USH2 complex members usherin and Whrnb, suggesting that Adgrv1 interacts with usherin and Whrnb in zebrafish photoreceptors. When comparing *adgrv1^rmc22^* zebrafish with wild-type controls, we furthermore observed increased levels of aberrantly localized rhodopsin in the photoreceptor cell body, and decreased electroretinogram (ERG) B-wave amplitudes which indicate that the absence of Adgrv1 results in impaired retinal function. Based on these findings we present the *adgrv1^rmc22^* zebrafish as the first *ADGRV1* mutant model that displays an early retinal dysfunction. Moreover, the observed phenotypic changes can be used as quantifiable outcome measures when evaluating the efficacy of future novel therapeutic strategies for *ADGRV1-*associated RP.

## 1. Introduction

Usher syndrome is an autosomal recessively inherited disorder characterized by hearing impairment and a progressive loss of visual function due to retinitis pigmentosa (RP). Depending on the clinical type, patients may also experience balance problems as a consequence of vestibular dysfunction. Currently, four types of Usher syndrome (USH1-4) are distinguished based on the onset, the severity and the progression of the clinical features [1]. Pathogenic variants in the *ADGRV1* gene, previously named *MASS1*, *VLGR1* or *GPR98*, have been identified as the underlying cause for Usher syndrome type 2C (USH2C) [2]. This type of Usher syndrome is characterized by congenital hearing impairment and a progressive loss of vision in the absence of vestibular dysfunction [3]. Worldwide, around 40,000 individuals develop USH2C, and these patients may benefit from hearing aids or cochlear implants to alleviate their hearing loss. However, there are currently no treatment options available to compensate for *ADGRV1-*associated loss of vision.

The *ADGRV1* gene encodes the largest cell surface protein known in humans: the adhesion G-protein-coupled receptor v1 (ADGRV1) [4]. The protein consists of a very large extracellular tail containing a signal peptide, multiple calx-beta domains, an epilepsy-associated repeat (EAR) domain, a thrombospondin/pentraxin/laminin G-like domain and a GPCR proteolytic site (GPS). A seven transmembrane region anchors the protein in the cell membrane, followed by a short cytoplasmic region containing a C-terminal class I PDZ binding motif [2,4,5].

In the retina, ADGRV1 co-localizes with usherin (USH2A) and whirlin (USH2D), and together these proteins shape the USH2 complex at the periciliary membrane of the photoreceptor cells [6]. Two types of photoreceptor cells can be distinguished, i.e., rods and cones, which are both built up by an outer segment (OS), an inner segment (IS), a cell body and a synaptic terminal. The OS contains the proteins responsible for phototransduction, and is separated from the IS by a connecting cilium. The membrane of the periciliary region, where the USH2 complex is localized, forms a collar-like extension of the IS that surrounds the connecting cilium. It has been shown that the USH2 complex forms molecular links between the periciliary membrane and the membrane of the connecting cilium [6]. Moreover, it has been proposed that the USH2 complex participates in the trafficking and docking of trans-Golgi-derived vesicles that contain components essential for OS function and maintenance, from the IS towards the OS [6,7,8,9]. Furthermore, for ADGRV1 specifically, it has been postulated that the protein is involved in the regulation of the calcium homeostasis at mitochondria-associated ER membranes, and that it is involved in cell spreading and migration by mechanosensing at focal adhesions [8,9]. However, the exact pathophysiological mechanism underlying *ADGRV1*-associated RP remains elusive.

To further unravel the pathophysiological mechanism underlying *ADGRV1*-associated RP, and to aid the development of future therapeutic strategies, suitable cellular or animal models that mimic the human phenotype are essential. Patient-derived cellular models such as fibroblasts or induced pluripotent stem cell (iPSC)-derived organoids provide the opportunity to perform studies in the patient’s own genetic and molecular context [10,11,12]. However, vision is a complex process that relies both on the conversion of light into electrical stimuli in the retina, and the integration and interpretation of these signals by the central nervous system. As such, animal models are still essential to evaluate the effect of therapies at the level of visual function. Multiple *Adgrv1* mouse models, either naturally occurring or genetically modified, have been reported. These models, however, only partially resemble the human phenotype since they do present with hearing loss, but do not recapitulate the retinal phenotype observed in patients [13,14,15,16,17,18]. This may be explained by interspecies anatomical differences in the photoreceptor periciliary region where Adgrv1 is expressed. It has been previously reported that the photoreceptor periciliary region is largely underdeveloped in rodents when compared to humans [19], making rodents less suitable to study *ADGRV1-*associated RP. As an alternative, zebrafish have been recognized as an attractive model to study retinal dysfunction [20,21,22]. Zebrafish have a retinal structure comparable to humans and also the photoreceptor periciliary region has been highly conserved. Moreover, the advantages of zebrafish such as their high fecundity, ex utero fertilization, rapid development and transparency of embryos and larvae allow for easy genetic manipulation and experimentation.

The introduction of CRISPR/Cas-based genome editing technologies significantly improved the possibilities of generating mutant zebrafish models in a time- and cost-effective manner [23]. In recent years several CRISPR/Cas9-based zebrafish mutants have been generated to study retinal dysfunction caused by pathogenic variants in the *USH2A* gene—the gene encoding usherin. In contrast to murine models, these mutant zebrafish models (*ush2a^rmc1^*, *ush2a^b1245^*, *ush2a^hzu6^* and *ush2a^u507^*) present with retinal dysfunction, already observed in larval stages, although in the absence of an obvious audiologic phenotype [20,21,22].

With the need for an animal model to study *ADGRV1-*associated RP, and with zebrafish being recognized as an attractive model to study retinal dysfunction, we here generated and characterized the *adgrv1^rmc22^* zebrafish model. In the zebrafish genome, *adgrv1* is present as a single copy that, as with the human *ADGRV1* gene, consists of 90 exons. By employing CRISPR/Cas9-based genome editing technology, we introduced protein-truncating lesions into *adgrv1*, resulting in the absence of Adgrv1 and the reduced expression of the USH2 complex members at the periciliary region of the *adgrv1^rmc22^* photoreceptors. Functional analyses revealed that knocking out *adgrv1* resulted in an early-onset retinal phenotype. Therefore, we present the *adgrv1^rmc22^* mutant as the first animal model that can be used to further unravel the molecular function of ADGRV1 in the retina, and to evaluate future therapeutic strategies for *ADGRV1*-associated RP.

## 2. Materials and Methods

### 2.1. Zebrafish Maintenance and Husbandry

Wild-type AB-strain zebrafish and *adgrv1^rmc22^* mutant zebrafish were bred and maintained by standard methods at the Radboud Zebrafish Facility Nijmegen [24]. Zebrafish eggs were obtained from pair-wise matings of wild-type and mutant zebrafish. Experimental procedures were conducted in accordance with international and institutional guidelines (protocol #RU-DEC, AVD10300202215892).

### 2.2. CRISPR/Cas9 Genome Editing Design and Microinjections

For the generation of *adgrv1^rmc22^* mutant zebrafish, CRISPR targets were identified and guide RNAs with settings for Cas9 were designed using the web tool CRISPRscan [25]. A cut-off value of at least 4 mismatches for potential off-target regions was used for the selection of an sgRNA. Based on the CRISPRscan predictions, an Alt-R^TM^ CRISPR/Cas9 sgRNA was ordered from Integrated DNA Technologies (IDT) for the following 20 bp genomic target sequence in *adgrv1* exon 9: 5′-GGGTATTCAGAGTCAGCCAG-3′.

The sgRNA and commercial Alt-R^®^ S.p. Cas9 Nuclease V3 (IDT, Coralville, IA, USA, #1081059) were co-injected in wild-type zebrafish embryos at the single cell stage. For this purpose, an injection mixture of 100 ng/μL sgRNA, 800 ng/μL Cas9 Nuclease, 300 mM KCl and 20% (*v*/*v*) phenol red solution (#P0290, Sigma Aldrich, Amsterdam, The Netherlands) was prepared and incubated at 37 ℃ for 5 min to allow sgRNA-Cas9 ribonucleoprotein complex formation. An amount of 1 nL of this mixture was injected into single-cell-stage zebrafish embryos using a Pneumatic PicoPump pv280 (World Precision Instruments, Friedberg, Germany). Following the injection, the embryos were raised at 28.5 °C in E3 embryo medium consisting of 5 mM NaCl, 0.17 mM KCl, 0.33 mM CaCl_2_, 0.33 mM MgSO_4_ and 0.1% (*v*/*v*) methylene blue. At 1 day post fertilization (dpf), 16 out of 200 injected embryos were analyzed for the presence of genome editing events using High-Resolution Melting Analysis (HRM) [26]. Upon detection of genome-editing HRM signatures, the remainder of the injected embryos were raised to adulthood (F0 fish). Founder fish that transmitted germline mutations were outcrossed twice with wild-type zebrafish to further minimize the possible presence of unforeseen CRISPR/Cas9-induced off-target modifications. After the second round of outcrossing, heterozygous fish were incrossed to produce progeny of which the homozygous fish and their wild-type siblings were used for subsequent breeding and phenotypic analysis.

### 2.3. High-Resolution Melting Analysis

At 1 dpf, injected and uninjected zebrafish embryos were sampled individually in 25 μL hotshot lysis buffer (25 mM NaOH, 0.2 mM EDTA), and incubated at 95 °C for 20 min. The lysates were neutralized with 2.5 μL 1 M Tris pH8, diluted 5–10× and used as input for high-resolution melting (HRM) PCR analysis, which was performed in a QuantStudio™ 3 Real-Time PCR system (Applied Biosystems, Waltham, MA, USA) using Q5^®^ High-Fidelity DNA Polymerase (New England Biolabs, Ipswich, MA, USA, #M0491L) and 1× EvaGreen Dye (Biotium, Fremont, CA, USA, #31000). Primers that were used for the PCR amplification are listed in Table S1. The HRM procedure was initiated after the PCR amplification by rapid cooling (4 °C/s) to 10 °C and a melt curve procedure (0.1 °C/s) during which data were collected. When typical HRM heteroduplex peaks were observed in >25% of the injected embryos (*n* = 16), the remainder of the injected embryos were raised to adulthood. Sanger sequencing was used to verify genome editing events in samples indicating heteroduplex HRM peaks.

### 2.4. Genotyping

Zebrafish samples, either individual 1 dpf embryos or adult tail fins, were lysed in 25 μL (embryos) or 75 μL (adult tail fins) lysis buffer (25 mM NaOH, 0.2 mM EDTA), and incubated at 95 °C for 20 min. The lysates were neutralized with 2.5 μL or 7.5 μL 1 M Tris pH 8, respectively, diluted 5–10× and used as input for PCR. The genomic region of *adgrv1* exon 9 surrounding the introduced lesion was amplified using standard PCR cycling conditions. Primers that were used for the PCR amplification are listed in Table S1. Amplicons and genotypes were confirmed using Sanger Sequencing.

### 2.5. Transcript Analysis

The total RNA was isolated from three pooled (in liquid nitrogen snap-frozen) larval heads of the same genotype. Larval heads were homogenized prior to lysis and RNA isolation was performed using the RNeasy Micro kit according to the manufacturer’s instructions (Qiagen, Hilden, Germany). Subsequently, 100 μg of the total RNA was used as a template for cDNA synthesis using the Superscript^TM^ IV Reverse Transcriptase kit (Thermo Fisher Scientific, Waltham, MA, USA, #18090200). An *adgrv1* fragment spanning exon 7 to exon 11 (1500 bp) was amplified using Q5^®^ High-Fidelity DNA Polymerase kit (New England Biolabs, Ipswich, MA, USA, #M0491L). An amplicon of *rpl13* was used as a reference. Cycling conditions were as follows: 98 °C for 1 min, 35 cycles of 98 °C for 10 s, 68 °C for 20 s and 72 °C for 2 min, followed by a final 72 °C for 5 min. Afterwards, amplicons were separated on a 2% agarose gel and analyzed using Sanger Sequencing. All primers used in transcript analyses are listed in Table S1.

### 2.6. Gene Expression Analysis

Following the same procedure as described in paragraph 2.5, the total RNA was isolated and cDNA was generated from four pools of five larvae (5 dpf), and from two juvenile zebrafish retinas (3 months post fertilization (3 mpf)) per genotype. Quantitative PCR was performed using GoTaq qPCR Master Mix (Promega) according to the manufacturer’s protocol. Transcript-specific primers have been designed targeted against *adgrv1* spanning from exon 84 to exon 85, *ush2a* exon 55 to exon 56, and *whrnb* exon 2 to exon 4. A target against the reference gene *rpl13* was also included. Amplifications were performed with the QuantStudio^TM^ 3 Real-Time PCR system (Applied Biosystems, Waltham, MA, USA). PCR reactions were performed in duplo and relative gene expression levels compared to the reference gene *rpl13* were determined with the 2^−∆Ct^ method. Primers that were used are listed in Table S1.

### 2.7. Antibodies, Immunohistochemistry and Histology

Zebrafish larvae (5 dpf) and the eyes of juvenile zebrafish (3 mpf) (*adgrv1^rmc22^* mutants and strain-matched wild-types) were cryoprotected with 10% sucrose in PBS for 5 min prior to embedding in an OCT compound (Sakura, Alphen aan den Rijn, The Netherlands, Tissue-Tek, #4583). After the embedding procedure, samples were slowly frozen using liquid-nitrogen-cooled isopentane, and cryosections (7 μm) were prepared following standard protocols. Unfixed cryosections were permeabilized for 20 min with 0.01% Tween-20 in PBS and blocked for 30 min with blocking buffer (10% normal goat serum and 2% bovine serum albumin in PBS). Both primary and secondary antibodies were diluted in blocking buffer, and sections were incubated overnight at 4 °C with the primary antibodies, followed by rinsing 3 times for 10 min with PBS, and a 1 h incubation with the secondary antibodies together with DAPI (1:800; D1306; Thermo Fisher, Waltham, MA, USA). After a second round of rinsing (3 times for 10 min with PBS), cryosections were post-fixed using 4% paraformaldehyde for 10 min, followed by rinsing 3 times for 5 min with PBS. Finally, the cryosections were mounted with Prolong Gold Anti-fade (P36930; Thermo Fisher, Waltham, MA, USA). The following primary antibodies and dilutions were used: rabbit anti-usherin (1:1000; #DZ01481, Boster Bio, Pleasanton, CA, USA), rabbit anti-Adgrv1 (1:100; #DZ41032; Boster Bio), rabbit anti-Whrnb (1:750; #42690002, Cip98a; Novus Biologicals, Centennial, CO, USA) and mouse anti-centrin (1:500; #04-1624; Millipore, Darmstadt, Germany). Secondary antibodies (Alexa Fluor 568 goat anti-rabbit (Thermo Fisher, Waltham, MA, USA, #A-11011) and Alexa Fluor 488 goat anti-mouse (Thermo Fisher, Waltham, MA, USA, #A-11029)) were used in a 1:800 dilution. The slides were imaged using a Zeiss Axio Imager fluorescence microscope equipped with an AxioCam MCR5 camera (Zeiss, Jena, Germany). The intensity of the usherin and Whrnb immunofluorescence was measured using Fiji (v.1.51n). Based on the centrin immunostaining, the outer segment layer was isolated. Next, a mask was generated based on the centrin staining using the “Find Maxima” option (noise = 25) and dilated five times. Next, the centrin mask and the usherin/Whrnb layer were combined to locate the exact position of the usherin or the Whrnb staining. Subsequently, the “Find Maxima” (noise = 10) was used to identify the usherin/Whrnb staining within the centrin mask, and touching objects were separated using the watershed option. The resulting mask was dilated six times to measure the area surrounding the usherin and Whrnb immunofluorescence signal. Subsequently, this enables a correction for background noise when the maximum and minimum gray values of the identified regions were measured on the original image of the usherin/Whrnb immunofluorescence (Analyze Particles option: size = 0–180, pixel circularity = 0.00–1.00).

For the rhodopsin labeling, *adgrv1^rmc22^* zebrafish and strain-matched wild-type larvae were raised in transparent 10 cm Petri dishes under normal light conditions (300 lux white light in a 14/10 h day/night rhythm), and larvae were sampled at the morning of 6 dpf exactly 100 min after light exposure. Cryosections (7 μm) of 4% PFA fixed larvae were stained and imaged as published previously [27]. As a primary antibody mouse anti-rhodopsin (1:4000, Clone 4D2; NBP2-59690, Novus Biologicals, Centennial, CO, USA) was used with secondary Alexa Fluor 488 goat anti-mouse (Thermo Fisher, Waltham, MA, USA, #A-11029) combined with DAPI (1:8000; D1306; Thermo Fisher, Waltham, MA, USA). The slides were imaged using a Zeiss Axio Imager fluorescence microscope equipped with an AxioCam MCR5 camera (Zeiss, Jena, Germany). Subsequently, images were blinded and randomized, and rhodopsin mislocalization was quantified independently by two researchers, by manually scoring the number of photoreceptor cells with a clear rhodopsin signal in the photoreceptor cell body per retinal section.

To assess the morphology of juvenile (3 mpf) and adult (6 mpf) zebrafish retinas, the eyes of *adgrv1^rmc22^* mutants and strain-matched wild-types were dissected. Using 4% paraformaldehyde (PFA) in PBS, the eyes were fixed overnight at 4 °C, dehydrated in ascending methanol series and incubated overnight in 100% methanol. Afterwards the eyes were rehydrated in a descending methanol series to 0.1% PBS-Tween-20, followed by cryoprotecting with 10% sucrose in 0.1% PBS-Tween-20 for 15 min, and 30% sucrose in 0.1% PBS-Tween-20 for 1 h at room temperature. Next, the eyes were embedded in the OCT compound (Sakura, Alphen aan den Rijn, The Netherlands, Tissue-Tek, #4583), slowly frozen using liquid-nitrogen-cooled isopentane, and cryosections (7 μm) were prepared and stained with hematoxylin and eosin and analyzed using a Zeiss Axioscope light microscope (Zeiss, Oberkochen, Germany).

### 2.8. Electroretinogram (ERG) Recordings

ERG recordings were performed on juvenile (6–8 weeks post fertilization (wpf)) *adgrv1^rmc22^* zebrafish and wild-type siblings. Before performing the ERG measurements, fish were dark-adapted for at least 30 min. Fish were anesthetized using 0.016% tricaine methane sulfonate solution and the spinal cord was severed to stop the heartbeat. A Petri dish was filled with agarose gel, and a reference electrode was placed into the gel. Next, the zebrafish was placed in this Petri dish with the right eye facing the light source. Using a 25-gauge syringe needle, a small incision was made at the edge of the cornea of the right eye, in which the recording electrode filled with E3 medium (5 mM NaCL, 0.17 mM KCl, 0.33 mM CaCl_2_ and 0.33 mM MgSO_4_) was placed [28]. Two consecutive 100 ms white light stimuli with an intensity of ~550 lux were applied at an 8 s interval [20,28,29]. Subsequently, the response was amplified 10,000 times with a band-pass of 700-0.1 Hz and recorded with the Signal6.03 software (Cambridge Electronic Design Limited, Cambridge, England). A baseline correction was performed on the recordings, with the baseline signal being determined during a 50 ms timespan, as the average signal before the stimulus was given. The maximum B wave amplitude was calculated after taking the average response to the two light stimuli. All steps were performed under dim, red light.

### 2.9. Statistical Analyses

All statistical analyses were performed using PRISM software (v9.0.0). The software was used to check for normality, calculate average scores and perform unpaired two-tailed Student’s *t*-tests (for the usherin and Whrnb quantification, rhodopsin localization and ERG recordings).

## 3. Results

### 3.1. Generation of an Adgrv1 Zebrafish Mutant Model

**Figure 1:**
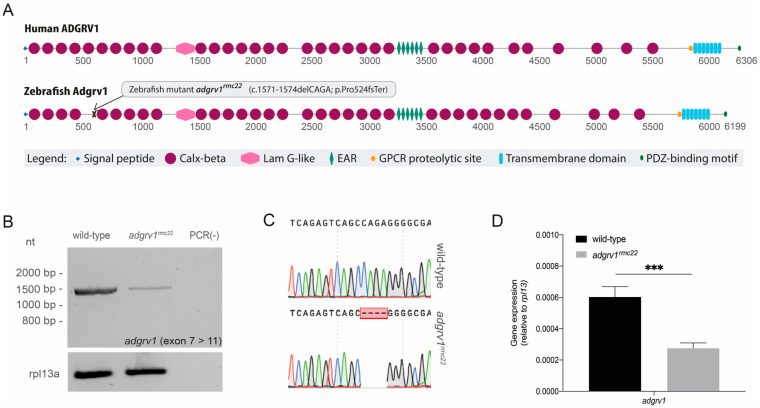
Domain architecture of human and zebrafish ADGRV1 and transcript analysis in homozygous *adgrv1^rmc22^* mutant zebrafish. (**A**): Schematic representation of human and zebrafish ADGRV1 protein domain structure. Both proteins have a similar repetitive protein domain architecture. Calx-beta: Ca^2+^-binding calcium exchanger beta domain; Lam G-like: thrombospondin/pentraxin/laminin G-like domain; EAR: epilepsy-associated repeats; GPCR: G protein-coupled receptor; PDZ: post-synaptic density 95, Discs large, Zonula occludens-1 binding motif. (**B**): RT-PCR analyses of *adgrv1* transcripts derived from homozygous *adgrv1^rmc22^* zebrafish larvae (5 dpf) and wild-type siblings revealed a reduction in mutant *adgrv1* transcripts when compared to wild-type *adgrv1* transcripts. (**C**): Sanger sequencing confirmed the four-base-pair deletion in the amplicon derived from homozygous *adgrv1^rmc22^* larvae. (**D**): RT-qPCR analysis of *adgrv1* transcripts in wild-type and *adgrv1^rmc22^* zebrafish larvae (5 dpf). Four pools of five larvae per genotype were used in RT-qPCR analysis. *** indicates *p* = 0.0001 (two-tailed unpaired Student’s *t*-test).

The conservation of the protein domain and gene architecture between human and zebrafish Adgrv1 was analyzed in order to identify a suitable CRISPR target region for the generation of *adgrv1* mutant zebrafish. Human and zebrafish Adgrv1 share a similar protein domain architecture (Figure 1A), and the multiple sequence alignment revealed a high degree of amino acid conservation (52.6% identity). Subsequently, an inventory of all protein-truncating pathogenic variants in human *ADGRV1* was made (*GPR98* LOVD mutation database, https://databases.lovd.nl/shared/genes/GPR98; 4 January 2021). Based on the presence of multiple protein-truncating variants (four unique loss-of function variants), exon 9 was selected as a target region. Using CRISPR/Cas9 genome editing technology we introduced a 4bp deletion in *adgrv1* exon 9 (c.1571-1574delCAGA; p.Pro524fsTer (ENSDART00000008043.9)). This frameshift mutation was predicted to result in the premature termination of translation, and the resulting zebrafish line was named *adgrv1^rmc22^*. The homozygous *adgrv1^rmc22^* mutants were viable, and no abnormalities in their development, morphology or swimming behavior were observed. Also, no obvious behavioral differences were observed that suggested a diminished auditory function [30]. Transcript analysis on the homozygous larvae was performed to visualize the effect of the 4bp deletion on *adgrv1* pre-mRNA splicing. Amplification of an amplicon spanning from *adgrv1* exon 7 to exon 11 on the larval mRNA did not reveal any alternative splicing events (Figure 1B), and Sanger sequencing of the *adgrv1^rmc22^* amplicon confirmed the 4bp deletion at the transcript level (Figure 1C). The lower intensity of the PCR product for the *adgrv1^rmc22^* sample could be indicative of the nonsense-mediated decay of mutant *adgrv1* transcripts. Subsequent RT-qPCR analyses confirmed that the relative expression of *adgrv1* was significantly lower in the mutant sample (*p* = 0.0001, two-tailed unpaired Student’s *t*-test) (Figure 1D).

### 3.2. Adgrv1 Is Absent from the Adgrv1^rmc22^ Photoreceptor Periciliary Region

**Figure 2:**
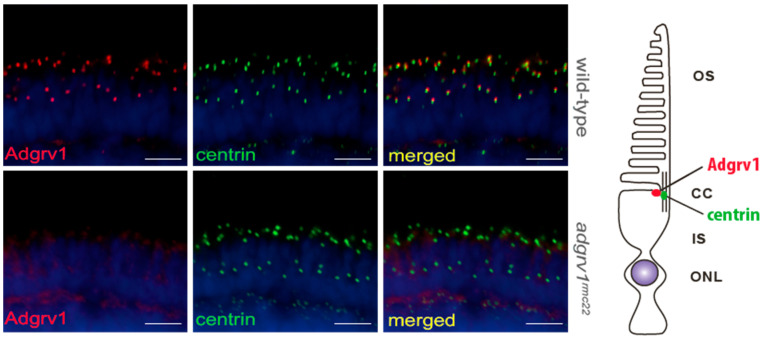
Localization of Adgrv1 in retinal cryosections of wild-type and *adgrv1^rmc22^* zebrafish. Retinal cryosections of wild-type and *adgrv1^rmc22^* zebrafish larvae (5 dpf) labeled with antibodies directed against Adgrv1 (red) and centrin (green) (as shown by the schematic representation on the right). Nuclei are counterstained with DAPI (blue). In wild-type larvae, Adgrv1 was detected adjacent to the connecting cilium marker centrin, whereas in *adgrv1^rmc22^* zebrafish no Adgrv1 signal could be detected at this location. Scale bar: 10 μm. OS: outer segment; CC: connecting cilium; IS: inner segment; ONL: outer nuclear layer.

In order to evaluate the effect of the introduced genomic lesion in *adgrv1* exon 9 on the expression and subcellular localization of Adgrv1, immunohistochemical analysis was performed on retinal cryosections of 5 dpf *adgrv1^rmc22^* zebrafish and strain-matched wild-type larvae, using an antibody directed against the N-terminal region of the Adgrv1 protein. Since Adgrv1 was previously shown to localize at the periciliary region adjacent to the connecting cilium [20], anti-centrin was used as a connecting cilium marker. In wild-type larvae, Adgrv1 indeed localized in close proximity to the photoreceptor connecting cilium; however, in retinal sections of *adgrv1^rmc22^* zebrafish larvae, the Adgrv1 protein could not be detected at this location (Figure 2). Immunohistochemical analysis on *adgrv1^rmc22^* and wild-type juvenile zebrafish retinas (3 mpf) corroborated these results (Figure S1A).

### 3.3. Absence of Adgrv1 Affects the Localization of USH2 Complex Members Usherin and Whrnb

**Figure 3:**
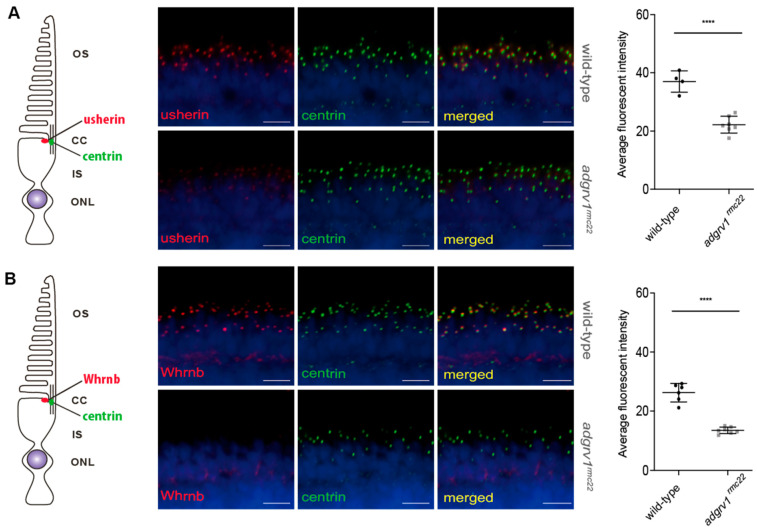
Reduced expression of usherin and Whrnb at the photoreceptor periciliary region of *adgrv1^rmc22^* zebrafish larvae. Retinal cryosections of wild-type and *adgrv1^rmc22^* zebrafish larvae (5 dpf) stained with antibodies directed against usherin (red) (**A**) or Whrnb (red) (**B**) and centrin (green). Nuclei are counterstained with DAPI (blue). (**A**): In wild-type larvae, usherin was present at the photoreceptor periciliary region in close proximity to the connecting cilium marker centrin. The intensity of the usherin signal in *adgrv1^rmc22^* retinal sections was significantly reduced when compared to wild-types (*n* = 7 *adgrv1^rmc22^* mutant larvae and *n* = 4 wild-type larvae). (**B**): In wild-type larvae, Whrnb was present at the photoreceptor periciliary region in close proximity to the connecting cilium marker centrin. The intensity of the Whrnb signal in *adgrv1^rmc22^* retinal sections was significantly reduced when compared to wild-types (*n* = 7 *adgrv1^rmc22^* mutant larvae and *n* = 6 wild-type larvae). Intensities of fluorescence signals were quantified (mean ± SD) and plotted in a scatter plot next to the corresponding pictures. **** indicates *p* < 0.0001 (two-tailed unpaired Student’s *t*-test). Scale bar: 10 μm.

Since it was proposed that Adgrv1 interacts with the other USH2 complex members in the zebrafish retina [20], immunohistochemical analyses were performed to evaluate the effect of the loss of Adgrv1 on the composition and localization of the USH2 complex members. Based on a previous study, it was hypothesized that the extracellular tail of Adgrv1 associates with the extracellular tail of usherin, whereas the intracellular region of Adgrv1 predominantly interacts with Whrnb [20]. Immunohistochemical analyses were performed on retinal cryosections of 5 dpf *adgrv1^rmc22^* zebrafish and strain-matched wild-type larvae, using antibodies directed against usherin and Whrnb (Figure 3). Both proteins were detected at the photoreceptor periciliary region in retinas of wild-type larvae, adjacent to the connecting cilium marker centrin. However, a strong reduction in the fluorescence signal of both usherin and Whrnb was observed in the retinas of 5 dpf *adgrv1^rmc22^* zebrafish. This was confirmed by quantification of the signal intensities (*p* < 0.0001, two-tailed unpaired Student’s *t*-test). Immunohistochemical analysis on juvenile *adgrv1^rmc22^* zebrafish retinas (3 mpf) confirmed the reduced localization of usherin and Whrnb also at later ages (Figure S1B,C). Both in larvae and in juveniles, there was no obvious usherin and Whrnb immunoreactivity visible elsewhere in the photoreceptors. This raises the question of whether the reduced immunoreactivity is the result of the disassembly of the USH2 complex, or is caused by altered gene expression levels. Therefore, RT-qPCR analyses were performed to study the relative *ush2a* and *whrnb* gene expression levels in larvae and juveniles (Figure S2). Mutant *adgrv1^rmc22^* larvae showed a 34% reduction in *ush2a* transcripts and a 26% reduction in the number of *whrnb* transcripts when compared to wild-types, whereas in *adgrv1^rmc22^* juveniles a reduction of 9% and 39% for *ush2a* and *whrnb*, respectively, was observed.

### 3.4. Defective Rhodopsin Transport in Adgrv1^rmc22^ Photoreceptor Cells

**Figure 4:**
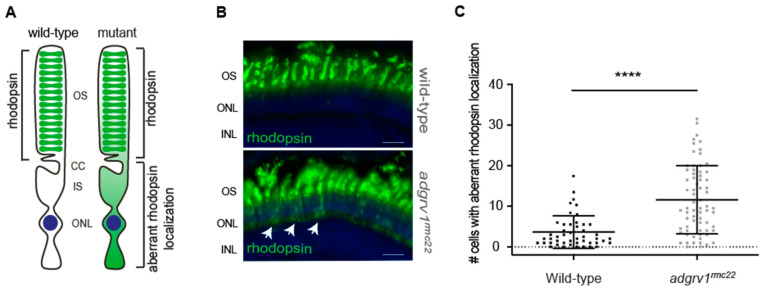
Aberrant localization of rhodopsin in photoreceptor cell bodies in the *adgrv1^rmc22^* zebrafish. (**A**): Schematic representation of a photoreceptor with rhodopsin localization in the outer segments (OS) versus aberrant rhodopsin localization in the photoreceptor cell body as observed in *adgrv1^rmc22^* mutants. (**B**): Retinal cryosections of wild-type and *adgrv1^rmc22^* zebrafish larvae (6 dpf) labeled with antibodies directed against rhodopsin (green). Nuclei are counterstained with DAPI (blue). A significantly higher number of photoreceptors with aberrant localization of rhodopsin was observed in *adgrv1^rmc22^* larvae (indicated with the white arrows) than in wild-type larvae. (**C**)**:** Total number of cells with aberrant rhodopsin localization per retinal section were plotted, with mean ± SD (*n* = 29 *adgrv1^rmc22^* mutant larvae and *n* = 21 wild-type larvae). A two-tailed unpaired Student’s *t*-test revealed a significant difference between *adgrv1^rmc22^* mutants and wild-types. **** indicates *p* < 0.0001, scale bar: 10 μm. CC: connecting cilium; IS: inner segment; ONL: outer nuclear layer; INL: inner nuclear layer.

Loss of USH2 complex proteins was previously linked to the impaired trafficking of rhodopsin in the photoreceptors of different animal models [21,31]. We therefore set out to examine the localization of rhodopsin in the photoreceptor cells of both wild-type (*n* = 21 eyes) and *adgrv1^rmc22^* (*n* = 29 eyes) larvae (6 dpf). Immunohistochemical analysis using an anti-rhodopsin antibody revealed that rhodopsin is located in the photoreceptor outer segments in the retinas of wild-type larvae. Occasionally, rhodopsin was also observed in the cell body of wild-type photoreceptors, which is in line with previous research [21,31]. However, the number of photoreceptors with aberrant localization of rhodopsin to the photoreceptor cell body was significantly higher in the retinal sections of *adgrv1^rmc22^* larvae than in wild-types (*p* < 0.0001, two-tailed unpaired Student’s *t*-test) (Figure 4). This result implies that the absence of Adgrv1 contributes to the aberrant ciliary trafficking of rhodopsin.

### 3.5. Adgrv1^rmc22^ Zebrafish Display Reduced Visual Function

**Figure 5:**
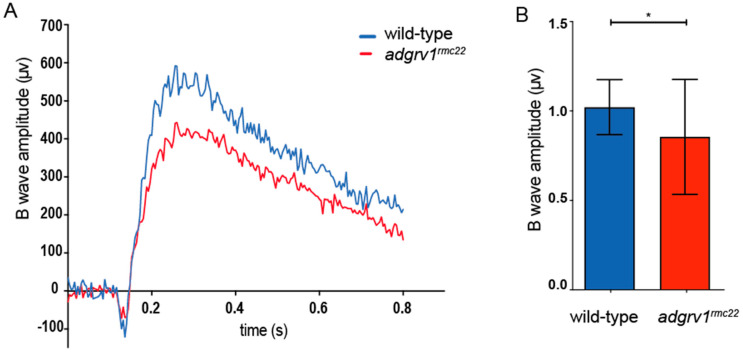
Electroretinogram recordings reveal impaired retinal function in *adgrv1^rmc22^* juveniles. (**A**): Representative ERG traces of an *adgrv1^rmc22^* and a wild-type zebrafish. (**B**): The *adgrv1^rmc22^* zebrafish show a significant decrease in maximum B-wave amplitude when compared to wild-type zebrafish (* *p* < 0.01, two-tailed unpaired Student’s *t*-test). ERG traces were recorded on the eyes of juvenile zebrafish (*n* = 35 *adgrv1^rmc22^* mutants and *n* = 31 wild-types, 6–8 weeks post fertilization). The average wild-type B-wave amplitude was normalized to 1. Mean B-wave amplitude ± SD is plotted in the bar graph.

Finally, electroretinogram (ERG) traces were recorded on *adgrv1^rmc22^* juveniles (6–8 wpf, *n* = 35) and age- and strain-matched wild-types (*n* = 31) to assess whether the absence of Adgrv1 at the photoreceptor periciliary region has implications for visual function. A significant decrease in the maximum B-wave amplitudes (16%) was observed in *adgrv1^rmc22^* mutants when compared to wild-type siblings (*p* = 0.011, two-tailed unpaired Student’s *t*-test) (Figure 5), which is indicative for the impaired visual function of *adgrv1^rmc22^* zebrafish.

## 4. Discussion

As a consequence of mutations in the *ADGRV1* gene, patients with Usher syndrome type 2C present with congenital hearing impairment and a progressive loss of vision due to RP. In the absence of a therapeutic strategy to prevent *ADGRV1-*associated RP, a suitable model that resembles the human retinal phenotype is essential to unravel the exact pathophysiological mechanism and to evaluate future therapeutic strategies. In this study, we generated and characterized the *adgrv1^rmc22^* zebrafish as a model for *ADGRV1-*associated retinal dysfunction. By employing CRISPR/Cas9 genome editing technology, we introduced a 4bp deletion in zebrafish *adgrv1* exon 9. We have shown that the *adgrv1^rmc22^* allele results in the absence of Adgrv1 at the photoreceptor periciliary region where its absence causes the disintegration of the USH2 protein complex. Moreover, an increase in the aberrant localization of rhodopsin was observed in *adgrv1^rmc22^* mutant larvae, and ERG recordings indicate reduced retinal function in juvenile *adgrv1^rmc22^* zebrafish. Based on these findings, we present the *adgrv1^rmc22^* zebrafish as a suitable model for studying *ADGRV1*-associated retinal dysfunction.

Until now, only *Adgrv1* mutant mouse models were reported, which in contrast to the *adgrv1^rmc22^* mutant zebrafish presented here, displayed an audiologic phenotype in the absence of a clear retinal phenotype [13,14,15,16,17,18]. In recent years, however, zebrafish have been recognized as suitable models to study *USH2A*-associated retinal dysfunction [20,21,22]. Due to the ex utero fertilization, zebrafish can be easily subjected to genetic manipulation and the introduction of CRISPR/Cas9 technology has radically reduced the efforts to generate targeted knock-out models when compared to ZFN- and TALEN-based genome editing [23,32,33]. To fill the gap in animal models to study *ADGRV1-*associated retinal dysfunction, we generated the CRISPR/Cas9-induced *adgrv1^rmc22^* mutant zebrafish.

Immunohistochemical analysis confirmed the presence of the USH2 proteins Adgrv1, usherin and Whrnb at the region of the connecting cilium in the wild-type zebrafish retina, which is in line with previous studies [20,21,31]. We showed that the introduction of a protein-truncating mutation at the N-terminal region of *adgrv1* resulted in the absence of the protein at the periciliary region of photoreceptor cells. Thus far, the effect of Adgrv1 depletion in the zebrafish retina has only been studied after morpholino antisense oligonucleotide-induced knock-down. In their study, however, Ebermann, et al. [34] did not report any retinal defects after the knock-down of *adgrv1*. When comparing our *adgrv1^rmc22^* zebrafish with the *adgrv1* knock-down larvae, the differences observed in retinal phenotype may be attributed to the transient and partial knock-down induced by morpholino antisense oligonucleotides versus the permanent protein-truncating mutation induced by CRISPR/Cas9. Additional immunohistochemical analyses revealed that the absence of Adgrv1 in the *adgrv1^rmc22^* zebrafish retina also results in a strong reduction in the usherin and Whrnb fluorescence signal. These results confirm the interdependency of these proteins in the correct formation of the periciliary USH2 protein complex that we previously proposed based on similar results in the *ush2a^rmc1^ and ush2a^b1245^* zebrafish [20]. Therefore, our findings confirm the proposed model in which Adgrv1 interacts in a protein complex with the other USH2 proteins usherin and Whrnb at the zebrafish photoreceptor periciliary region. To unravel if the reduced immunoreactivity of usherin and Whrnb at this region is the result of the disassembly of the USH2 complex, or caused by altered gene expression levels, we analyzed the *ush2a* and *whrnb* gene expression levels. In *adgrv1^rmc22^* mutant larvae, the relative gene expression levels show a statistically significant reduction of 34% and 26% for *ush2a* and *whrnb*, respectively. It has been described that as little as 20% of functional *ush2a* transcripts can be sufficient to result in usherin localization at the zebrafish photoreceptor periciliary region [35]. Therefore, it is likely that the reduced localization of usherin and Whrnb in the *adgrv1^rmc22^* zebrafish retina is the consequence of the absence of Adgrv1 resulting in the disintegration of the USH2 complex.

The USH2 protein network is hypothesized to contribute to the ciliary trafficking of cargo, such as rhodopsin, towards the photoreceptor outer segments. It is also well accepted that the accumulation of proteins in the inner segments due to the dysfunction of ciliary transport may underlie the photoreceptor degeneration through the activation of cell stress pathways [6,36]. Moreover, it was reported that mislocalized rhodopsin results in ectopic phototransduction [37], a process which has been found to accelerate photoreceptor cell death [38]. The significant increase in the number of photoreceptors with the aberrant localization of rhodopsin in the retinal sections of *adgrv1^rmc22^* larvae indeed supports the notion that defective ciliary protein transport may lie at the origin of *ADGRV1*-associated retinal degeneration. Interestingly, histological examination of the juvenile (3 mpf) and adult (6 mpf) *adgrv1^rmc22^* zebrafish retina did not reveal any signs of progressive retinal degeneration (Figure S3), as the *adgrv1^rmc22^* zebrafish retinal layers were morphologically indistinguishable from wild-type retinas. The absence of a clear progressive phenotype, however, is in line with the previously published *ush2a* mutant zebrafish models, where also no morphological differences were observed when compared to wild-types [20]. A plausible explanation for the absence of the progressive degeneration of photoreceptor cells lies in the regenerative capacity of the zebrafish retina, since in contrast to the human retina, the zebrafish retina is able to replace lost retinal neurons [39]. The phototoxic effect of mislocalized rhodopsin resulting in photoreceptor cell death may thus be masked by the continuous photoreceptor regeneration. Lacking a progressive phenotype, the restoration of the USH2 protein complex at the periciliary region, and normal rhodopsin trafficking, provide valuable biomarkers to evaluate future therapeutic strategies for *ADGRV1*-associated retinal degeneration in the *adgrv1^rmc22^* zebrafish.

In the current study, we assessed the function of Adgrv1 localized at the photoreceptor periciliary membrane. Interestingly, a recent paper identified ADGRV1 at the ER membrane and at the mitochondria-associated ER membranes (MAMs) in cells derived from VLGR1-deficient mouse models, suggesting a novel function for ADGRV1 [9]. The absence of ADGRV1 at these internal membranes results in increased distances between the ER and mitochondria, and in reduced Ca^2+^ release from the ER, thereby disrupting the bioenergetics of cells [9]. Due to the high energy demand of photoreceptor cells, it is tempting to speculate that these mechanisms could also underlie photoreceptor cell dysfunction [40]. Based on our immunohistochemical analysis, however, we obtained no indication that Adgrv1 is present at the ER membranes and MAMs in photoreceptor cells, as this requires high-resolution microscopy techniques.

Finally, ERGs were recorded in the eyes of juvenile *adgrv1^rmc22^* (6–8 wpf) mutants and wild-type siblings. At this age, zebrafish rod and cone photoreceptors are considered to be mature, and both contribute to the visual function [41]. When examining ERG traces, first a small initial A-wave is observed that represents the hyperpolarization of photoreceptor cells. The A-wave is largely obscured by the thereafter following B-wave that represents the depolarization of the ON bipolar cells [42]. ERG recordings demonstrate a significant decrease in B-wave amplitude in the *adgrv1^rmc22^* zebrafish when compared to wild-types. The decrease in B-wave amplitude could be a result of a defect in phototransduction or in signal transduction towards the ON bipolar cells. Either way, the significant decrease in B-wave amplitude in the *adgrv1^rmc22^* zebrafish can be considered as an indication of impaired retinal function. These results are in line with the previously published ERGs recorded on the eyes of *ush2a* mutant zebrafish larvae, where similar reductions in B-wave amplitudes were recorded [20,21,22,31].

## 5. Conclusions

In conclusion, we have generated an *adgrv1^rmc22^* mutant zebrafish model that, to our notice, is the first *ADGRV1* mutant animal that displays an early retinal dysfunction. Previous research has already proven the translational value of zebrafish as a model to study retinal dystrophies [20,41,43]. Moreover, studies by Schellens et al. (accepted) and Dulla et al. [35] have proven the relevance of zebrafish when evaluating therapeutic strategies such as single- or dual-exon skipping therapy for *USH2A-*associated RP. Specifically, the early molecular phenotypes that we observe in the photoreceptors of *adgrv1^rmc22^* larvae provide an unmatched opportunity to assess and optimize potential therapeutic strategies for *ADGRV1*-associated RP. The presented zebrafish model therefore brings us one step closer towards the future treatment of *ADGRV1-*associated RP.

## Data Availability

All data is available upon request.

## Supplementary Materials

The following supporting information can be downloaded at: https://www.mdpi.com/article/10.3390/cells12121598/s1, Figure S1. Reduced expression of Adgrv1, usherin and Whrnb at the photoreceptor periciliary region of *adgrv1^rmc22^* zebrafish. Retinal cryosections of wild-type and *adgrv1^rmc22^* zebrafish (3 mpf), stained with antibodies directed against Adgrv1 (red) (A), usherin (red) (B), or Whrnb (red) (C) and centrin (green). Nuclei are counterstained with DAPI (blue). In the wild-type retina, Adgrv1, usherin and Whrnb are present at the photoreceptor periciliary region in close proximity to the connecting cilium marker centrin. The intensity of the Adgrv1, usherin and Whrnb signal in *adgrv1^rmc22^* retinal sections is reduced when compared to wild types. Scale bar: 20 μm. Figure S2: RT-qPCR analysis of *ush2a* and *whrnb* transcripts in wild-type and *adgrv1^rmc22^* zebrafish. A: Four pools of 5 larvae (5 dpf) per genotype were used in RT-qPCR analysis. In the *adgrv1^rmc22^* mutant samples a 34% reduction of *ush2a* transcripts, and a 26% reduction of the *whrnb* transcripts is observed. * indicates *p* < 0.05 (two-tailed unpaired Student’s *t*-test). B: Two samples of two single juvenile (3 mpf) zebrafish retinas per genotype were used in RT-qPCR analysis. Although not significant, in the *adgrv1^rmc22^* mutant samples a 9% reduction of *ush2a* transcripts, and a 39% reduction of the *whrnb* transcripts is observed. ns: not significant (two-tailed unpaired Student’s *t*-test). Figure S3. Histological examination of wild-type and *adgrv1^rmc22^* zebrafish retinas. Retinal sections of wild-type and *adgrv1^rmc22^* zebrafish (3 mpf and 6 mpf) stained with hematoxylin (purple) and eosin (red). Retinas of wild-type and *adgrv1^rmc22^* zebrafish of the same age are morphologically indistinguishable. Scale bar: 20 μm. RPE: retinal pigment epithelium, ONL: outer nuclear layer, OPL: outer plexiform layer, INL: inner nuclear layer, GCL: ganglion cell layer. Table S1: oligo list.
