# Supplementary material for: Generation and Characterization of a Zebrafish Model for ADGRV1-Associated Retinal Dysfunction Using CRISPR/Cas9 Genome Editing Technology
Source: Cells. 2023 Jun 10;12(12):1598. doi: 10.3390/cells12121598 (PMC10296736; doi:10.3390/cells12121598)
Supplement: Supplementary file 1 [file cells-12-01598-s001.zip › cells-2327376-supplementary.pdf]

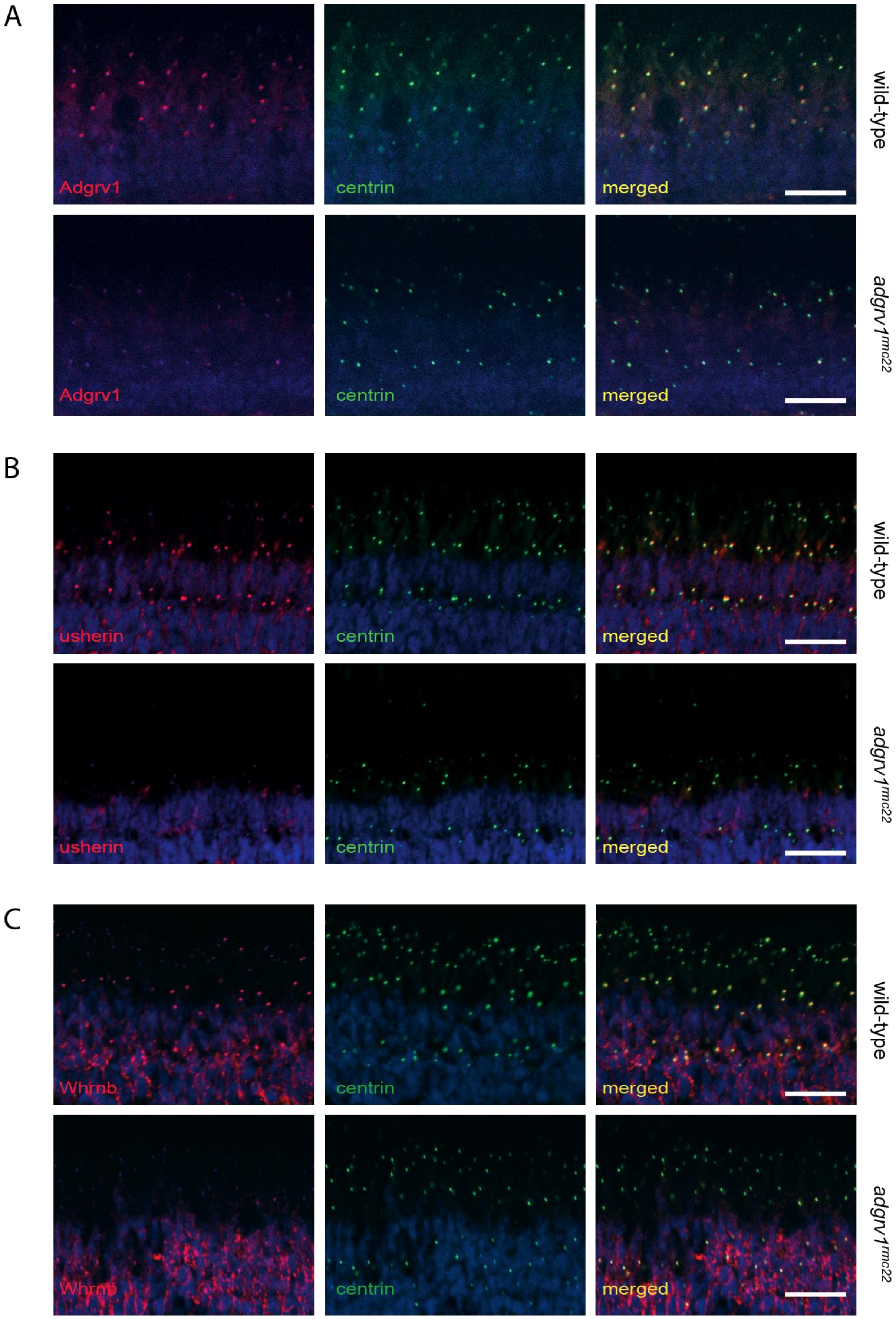
A
Adgrv1
centrin
merged
wild-type
adgrv1mmc22
B
usherin
centrin
merged
wild-type
adgrv1mmc22
C
Whrnb
centrin
merged
wild-type
adgrv1mmc22

Figure S1. Reduced expression of Adgrv1, usherin and Whrnb at the photoreceptor periciliary region of *adgrv1^{rmc22}* zebrafish. Retinal cryosections of wild-type and *adgrv1^{rmc22}* zebrafish (3 mpf), stained with antibodies directed against Adgrv1 (red) (Fig.A), usherin (red) (Fig.B), or Whrnb (red) (Fig.C) and centrin (green). Nuclei are counterstained with DAPI (blue). In the wild-type retina, Adgrv1, usherin and Whrnb are present at the photoreceptor periciliary region in close proximity to the connecting cilium marker centrin. The intensity of the Adgrv1, usherin and Whrnb signal in *adgrv1^{rmc22}* retinal sections is reduced when compared to wild types. Scale bar: 20 μm.

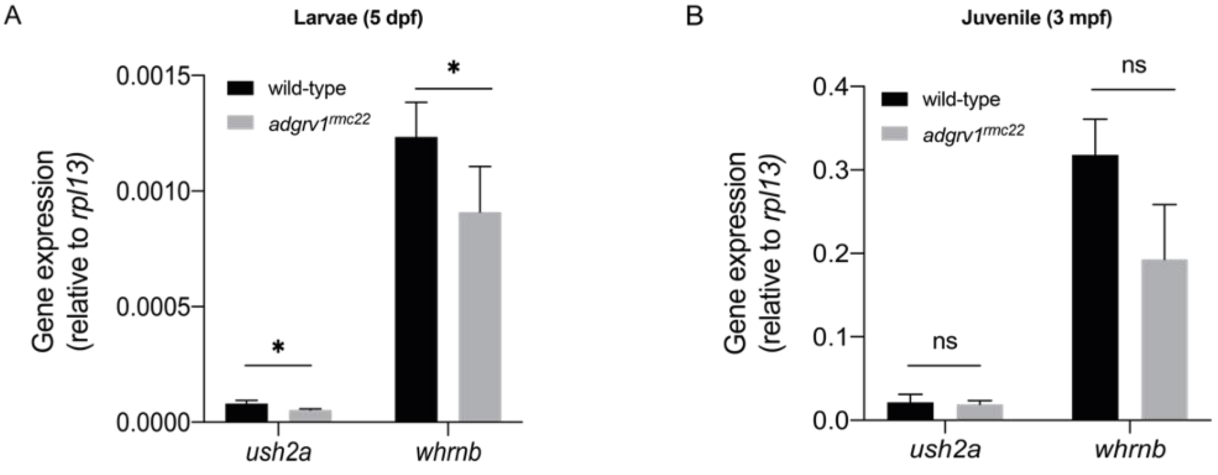

Figure S2: RT-qPCR analysis of *ush2a* and *whrnb* transcripts in wild-type and *adgrv1^{rmc22}* zebrafish. A: Four pools of 5 larvae (5 dpf) per genotype were used in RT-qPCR analysis. In the *adgrv1^{rmc22}* mutant samples a 34% reduction of *ush2a* transcripts, and a 26% reduction of the *whrnb* transcripts is observed. * indicates P < 0.05 and *** indicates P < 0.0005 (two-tailed unpaired Student's t-test). **B:** Two samples of two single juvenile (3 mpf) zebrafish retinas per genotype were used in RT-qPCR analysis. Although not significant, in the *adgrv1^{rmc22}* mutant samples a 9% reduction of *ush2a* transcripts, and a 39% reduction of the *whrnb* transcripts is observed. ns: not significant and ** indicates P < 0.005 (two-tailed unpaired Student's t-test).

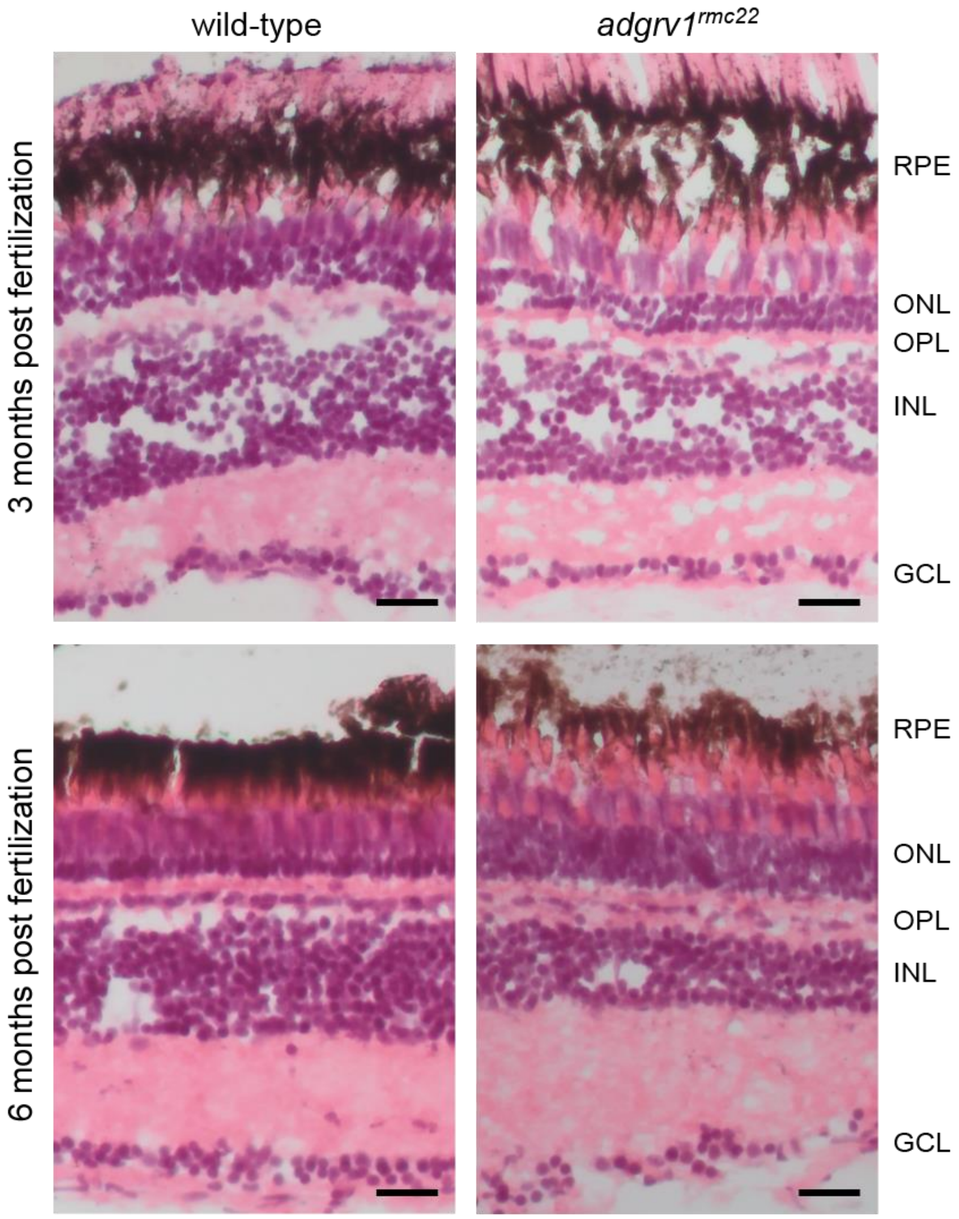

Figure S3. Histological examination of wild-type and *adgrv1^rmc22^* zebrafish retinas. Retinal sections of wild-type and *adgrv1^rmc22^* zebrafish (3 mpf and 6 mpf) stained with hematoxylin (purple) and eosin (red). Retinas of wild-type and *adgrv1^rmc22^* zebrafish of the same age are morphologically indistinguishable. Scale bar: 20 μm. RPE: retinal pigment epithelium, ONL: outer nuclear layer, OPL: outer plexiform layer, INL: inner nuclear layer, GCL: ganglion cell layer.

Table S1: oligo list

**Target:**	**Sequence (5′>3′)**
sgRNA *adgrv1* exon 9 (targeting sequence)	GGGTATTCAGAGTCAGCCAG
ZF_*adgrv1*_ex9_fw (HRM)	TATGGTCGATTTGGCTTTCATCC
ZF_*adgrv1*_ex9_rv (HRM)	GGGCAGTCAGTGTAAGTCGT
ZF_*adgrv1*_ex7_cDNA_Fw (RT-PCR)	GTGCAGATCAAGATCTCCCGT
ZF_*adgrv1*_ex11_cDNA_Rv (RT-PCR)	GTTGTCCACATTTGTCCTGTCT
ZF_*rpl13a*_household_cDNA_Fw	TCTGGAGGACTGTAAGAGGTATGC
ZF_*rpl13a*_household_cDNA_Rv	AGACGCACAATCTTGAGAGCAG
ZF_*adgrv1*_ex84_qPCR_Fw	AGGACAGTTGTGCTGCTCTC
ZF_*adgrv1*_ex85_qPCR_Rv	GCAGATACCGTCTCTCCGTG
ZF_*ush2a*_ex55_qPCR_Fw	CAGACTGGCGTTGGAGTCAT
ZF_*ush2a*_ex56_qPCR_Rv	GCTGTAGTTGGTGTGAGGCT
ZF_*whrnb*_ex2_qPCR_Fw	TGGGTCTGATGATCCGAGGT
ZF_*whrnb*_ex4_qPCR_Rv	GCCCACGTCCTTTATGGTCA
